# 1397. What were the Birth Outcomes of Infants Born to Zika Positive Mothers in Developing Countries?

**DOI:** 10.1093/ofid/ofad500.1234

**Published:** 2023-11-27

**Authors:** Monique A Prince, Lauren Orlando, E’ebony Prince, Abidemi Fasanmi

**Affiliations:** Saint Michael's Medical Center, Newark, New Jersey; St. George’s University, True Blue, Saint George, Grenada; St. George's University, True Blue, Saint George, Grenada; St. George’s University, True Blue, Saint George, Grenada

## Abstract

**Background:**

Zika Virus became an emerging health threat in the early 21^st^ century. Epidemics in the Pacific Islands noted flu-like symptoms and complications following human ZIKV infection. However, after a severe outbreak in 2015, Latin American studies reported fetal birth defects with maternal infection but reports in other developing countries where outbreaks occurred were not fully characterized. This study aims to review current reports on birth outcomes associated with maternal ZIKV infection .

**Methods:**

This narrative review evaluated birth outcomes of Zika positive pregnant women. Research articles were found using Google Scholar, PubMed, and MEDLINE. The inclusion criteria were publication between 2012 to 2022, availability as full text in English, categorization as original research, meta-analyses or case reports. Exclusion criteria were studies that were part of a book chapter or encyclopedia and studies that cited results from developed nations. The articles were studied and 20 methodologically sound studies were selected for review, and the data was classified in themes based on birth outcomes.

**Results:**

18 studies reported that infants born to some laboratory-confirmed zika-positive mothers had abnormal birth outcomes after delivery including microcephaly, stillbirth, neurological abnormalities and hearing loss. Maternal ZIKV infection is associated with an increased risk of abnormal birth outcomes in newborns in developing countries. In the countries of studies that did not report abnormal outcomes there may have been a protective factor from previous maternal Dengue Virus (DENV) infection adding protection for their infants.

**Conclusion:**

Overall, there appears a difference in birth outcomes of infants born to mothers infected with ZIKV, however more research is needed on environmental factors and prior arboviral infection as additional factors contributing to these outcomes.

**Disclosures:**

**All Authors**: No reported disclosures

